# Ultraprocessed Food Intake, Striatal Response to Food Cues, and Adiposity Gain in Children

**DOI:** 10.1001/jamanetworkopen.2026.40252

**Published:** 2026-10-23

**Authors:** Jasmin M. Alves, Ella Morgan, Brendan Angelo, Kianna Le, Jared Smith, Ting Chow, Kay Jann, Anny H. Xiang, Kathleen A. Page

**Affiliations:** 1 Division of Endocrinology and Diabetes, Department of Medicine, Keck School of Medicine, University of Southern California, Los Angeles; 2 Diabetes and Obesity Research Institute, Keck School of Medicine, University of Southern California, Los Angeles; 3 Department of Research and Evaluation, Kaiser Permanente Southern California, Pasadena; 4 University of Southern California Stevens Neuroimaging and Informatics Institute, Keck School of Medicine, University of Southern California, Los Angeles; 5 Department of Health Systems Science, Kaiser Permanente Bernard J. Tyson School of Medicine, Pasadena, California

## Abstract

**Importance:**

Preclinical studies suggest that ultraprocessed foods (UPFs) are associated with increased dopaminergic signaling in the striatum, reinforcing intake. Whether habitual UPF consumption is associated with striatal responses to food cues and subsequent adiposity gain in children is unknown.

**Objective:**

To examine associations between habitual UPF intake, striatal responses to food cues, and 1-year adiposity changes in children.

**Design, Setting, and Participants:**

This prospective cohort study included children of healthy weight who were participating in the longitudinal BrainChild study. Baseline assessments were completed between April 11, 2015, and June 28, 2024, and included brain magnetic resonance imaging (MRI), dietary recalls, and adiposity measurements. Additional follow-up assessments of adiposity were conducted 1 year later (April 18, 2016, to June 19, 2025).

**Exposure:**

Habitual UPF intake, defined as the percentage of total energy intake from UPFs based on multiple 24-hour dietary recalls using the NOVA classification system.

**Main Outcomes and Measures:**

Blood oxygenation level–dependent (BOLD) responses to food cues in the dorsal and ventral striatum during functional MRI and 1-year changes in body mass index (BMI), body fat percentage, and waist circumference. Associations were examined using linear regression models and adjusted for age, sex, baseline BMI, and total caloric intake, with regression coefficient (β) reported as per SD change in outcome with per SD change in the exposure variable.

**Results:**

In 46 children of healthy weight (mean [SD] age, 8.7 [1.1] years; 26 [57%] female; baseline BMI range, 13.8-19.9; body fat percentage range, 12.7%-32.2%), higher habitual UPF consumption was associated with greater BOLD responses to food cues in the dorsal striatum (β = 0.34; 95% CI, 0.07-0.61) but not the ventral striatum (β = 0.16; 95% CI, −0.14 to 0.46). Greater dorsal striatal food cue responsivity was associated with 1-year increases in BMI (β = 0.32; 95% CI, 0.02-0.62) and body fat percentage (β = 0.44; 95% CI, 0.15-0.72). Baseline UPF intake was associated with a 1-year increase in waist circumference (β = 0.38; 95% CI, 0.11-0.66). Point estimates for associations between UPF intake and increases in BMI (β = 0.28; 95% CI, 0.00-0.57) and body fat percentage (β = 0.29; 95% CI, 0.00-0.57) were positive. Inclusion of dorsal striatal responsivity attenuated the UPF associations with 1-year change in BMI by 31% and with body fat percentage by 44%.

**Conclusions and Relevance:**

In this cohort study of children of healthy weight, higher habitual UPF intake was associated with greater dorsal striatal responses to food cues and prospective increases in adiposity. These findings suggest that reward-related brain responses may be involved in associations between UPF consumption and adiposity gain during childhood.

## Key Points

**Question:**

Is habitual ultraprocessed food (UPF) intake during childhood associated with striatal responses to food cues, and are these brain responses associated with subsequent adiposity gain?

**Findings:**

This cohort study of 46 children of healthy weight found that higher habitual UPF intake was associated with greater dorsal striatal responses to food cues, which were, in turn, associated with greater adiposity gain over 1 year. The association between UPF intake and adiposity gain was attenuated after accounting for dorsal striatal responses.

**Meaning:**

These findings suggest that reward-related brain responses may contribute to the association between UPF intake and adiposity gain during childhood.

## Teaser

This cohort study examines associations between habitual intake of ultraprocessed foods and striatal responses to food cues, and between striatal responsivity and subsequent adiposity changes in children of healthy weight.

## Introduction

Ultraprocessed foods (UPFs) are calorically dense, nutrient-poor products that are heavily marketed to children and often formulated to be highly palatable and rewarding.^1,2^ On average, UPFs account for 67% of calories consumed by US children,^3^ and higher UPF consumption has been associated with unfavorable metabolic outcomes in both pediatric and adult populations.^4,5,6,7,8,9^ However, associations between UPF intake, longitudinal changes in adiposity, and the neurobiological pathways that might contribute to such associations in children remain unclear. Childhood is a sensitive period for the maturation of brain reward circuits and long-term energy balance regulation, underscoring the importance of understanding how dietary exposures may interact with neural systems during this developmental window and contribute to obesity risk.^10,11^

The striatum is a brain region that plays a prominent role in learning, motivation, and reward anticipation.^12^ Prior neuroimaging studies in adolescents and adults have shown that palatable food cues and food receipt elicit striatal responses,^13,14^ and that greater striatal responses to food cues are associated with larger increases in body weight and body fat over time.^15,16,17^ The incentive-sensitization theory of reward offers one framework for these observations, distinguishing dopamine-mediated incentive salience (“wanting”) from the hedonic experience of reward (“liking”). According to this theory, repeated exposure to highly rewarding stimuli progressively sensitizes neural circuits underlying wanting, leading to heightened striatal responses to reward predictive cues independent of hedonic value.^15^ Because UPFs are highly rewarding and often consumed repeatedly throughout childhood, habitual UPF intake may be associated with incentive sensitization, resulting in heightened striatal response to food cues. Whether UPF consumption is associated with striatal responsivity to food cues in children, and whether such brain responses are associated with subsequent adiposity gain, remains unknown.

A longitudinal study in children showed that higher UPF intake was associated with greater food responsiveness and larger increases in body mass index (BMI; calculated as weight in kilograms divided by height in meters squared) over 3 years, with the authors proposing that altered hedonic regulation may be one pathway linking UPF consumption with weight gain.^7^ Therefore, in a prospective cohort of children of healthy weight, we examined whether higher habitual UPF intake was associated with greater striatal responses to food cues and whether baseline striatal responsivity was associated with 1-year increases in adiposity. We hypothesized that children with higher UPF intake would have greater striatal responses to food cues and that greater striatal responsivity would be associated with larger increases in adiposity over 1 year.

## Methods

### Participant Overview

This prospective cohort study used data from the BrainChild Cohort,^16^ a longitudinal study of children to investigate how prenatal exposure to gestational diabetes is associated with neural and metabolic outcomes. Children were recruited at baseline (age, 7-10 years) from Kaiser Permanente Southern California based on gestational diabetes exposure status. Baseline assessments were completed between April 11, 2015, and June 28, 2024; additional follow-up assessments of adiposity were conducted 1 year later (April 18, 2016, to June 19, 2025). Inclusion criteria were the absence of preexisting health conditions in the children and the availability of complete maternal pregnancy information. Additional details on the BrainChild Cohort are available elsewhere.^17^ An additional inclusion criterion for this analysis was a healthy weight status at baseline (<85th BMI percentile) to minimize obesity as a potential confounder. Race and ethnicity (Hispanic or Latino, non-Hispanic Asian or Pacific Islander, non-Hispanic Black or African American, non-Hispanic White, non-Hispanic other [mixed race or ethnicity]) were self-defined by the child or parent; categories were investigator defined and are reported because dietary patterns and adiposity risk may differ across racial and ethnic groups. Pubertal development was assessed using Tanner staging, determined by physician examination and, when unavailable, by self-report. Upon recruitment, participants provided assent and their parents provided written consent. The institutional review boards at Kaiser Permanente Southern California and the University of Southern California (USC), where study visits were completed, approved this study. This study followed the Strengthening the Reporting of Observational Studies in Epidemiology (STROBE) reporting guideline for cohort studies.

### Study Visits

#### Adiposity Measurements

For an overview of study visits, see eFigure 1 in Supplement 1. Adiposity was assessed at baseline and at 1-year follow-up in the morning, after a 12-hour overnight fast, at the Diabetes and Obesity Research Institute Clinic of USC. Body fat percentage and body weight were measured using the Tanita Body Composition Analyzer SC-331S (Tanita Corporation of America Inc), which has been validated for the assessment of body composition in children.^18^ Height was measured using a stadiometer. Waist circumference in centimeters was measured using a measuring tape positioned at the midpoint between the iliac crest and the lower costal margin in the midaxillary line.

#### MRI Acquisition and Food Cue Task

Approximately 1 month after a baseline adiposity assessment, participants completed a magnetic resonance imaging (MRI) session between 8 and 10 am after a 12-hour overnight fast at the USC Dana and David Dornsife Cognitive Neuroimaging Center. Prior to the functional MRI (fMRI) scan, participants consumed a weight-dependent glucose beverage (1.75 g/kg body weight, maximum 75 g; Azer Scientific) to standardize satiety state, as striatal response to food cues varies significantly with hunger state.^19^ fMRI using T2-weighted blood oxygenation level–dependent (BOLD) scans (for sequence details, see below) were acquired in the supine position while participants performed the food-cue task, a randomized block design with 12 blocks alternating between food and nonfood visual cues. Each block presented 3 color photographs in random order for 4 seconds each, separated by a 1-second interstimulus interval, for a total run time of 3 minutes and 16 seconds. Participants viewed stimuli via a mirror mounted on the head coil and an MR-compatible monitor at the end of the bore. Food cues showed highly palatable food items such as cookies or French fries, while nonfood cues showed neutral objects such as a pencil or a bench. The task was programmed in MATLAB, version R2008a (The MathWorks Inc), and run using Psychtoolbox, version 3.0, on a Mac laptop. Regions of interest (ROIs) included the dorsal striatum (caudate and putamen) and ventral striatum (nucleus accumbens), defined anatomically using the Harvard-Oxford atlas.^20^ Detailed acquisition parameters and preprocessing procedures are described in the eMethods in Supplement 1.

### Seed to Voxel Psychophysiological Interaction Functional Connectivity Analysis

Exploratory generalized psychophysiological interaction analyses were performed using the CONN functional connectivity toolbox, version 21.a (Alfonso Nieto-Castanon), and SPM12, version 12.7771 (University College London), to assess functional connectivity between the striatal seed regions, the bilateral dorsal striatum, the bilateral ventral striatum, and other voxels of the brain during the food-cue task. These analyses characterized how striatal connectivity varied in association with UPF intake. For each seed, the mean BOLD time series was extracted across each bilateral seed ROI and used to estimate seed-to-voxel generalized psychophysiological interaction connectivity maps for the food vs nonfood contrast. Group-level effects were tested using voxelwise general linear models, with first-level connectivity estimates as dependent variables and participant-level factors as independent variables, including participant age, sex, and UPF intake. Random-effects analyses were conducted across participants, with inference performed at the cluster level. Results were thresholded at the voxel level of *P* < .05 and at the cluster level of family-wise error (FWE) *P* < .05, using the FWE rate to control for multiple comparisons. Additional processing details can be found in the eMethods in Supplement 1.

### Dietary Assessment and UPF Classification

At baseline visit, participants completed two 24-hour dietary recalls on separate days using the multipass method, conducted by trained staff with input from both the child and parent (eFigure 1 in Supplement 1). The 2 recalls were averaged for each participant. During each recall, participants were asked to recall the food and beverages they consumed within the past 24 hours. Using the multipass method, trained staff further reviewed the recall through several structured passes to probe for additional details, including preparation method, quantity consumed, condiments added, and brand name of food consumed, improving completeness and accuracy.^21,22^ Completed recalls were entered into the Nutritional Data System for Research software, version 2024 (Nutrition Coordinating Center, University of Minnesota). Please see the eMethods in Supplement 1 for additional quality control details.

#### NOVA Classification of Food Items

The NOVA classification system^23^ was used to categorize foods by processing level methods from Sneed et al.^24^ Foods were classified as 4 levels (1, unprocessed or minimally processed; 2, processed culinary ingredients; 3, processed foods; 4, UPFs). The primary exposure was the percentage of total daily energy intake derived from UPFs (NOVA level 4). For additional processing details, see eTable 1 in Supplement 1.

### Statistical Analysis

Descriptive statistics and linear regression were completed using SAS OnDemand for Academics, version 9.4 (SAS Institute Inc). Statistical figures were created in R Studio, version 2026.01.0 + 392 (R Project for Statistical Computing). All analyses were complete case. Of 244 BrainChild participants enrolled at baseline, 144 had follow-up data at year 1, and 75 had complete dietary, anthropometric, and brain imaging data for both baseline and year 1. Of those 75 participants, 46 had a healthy weight (<85th BMI percentile) at baseline and were included in the analysis. There were no missing data among the 46 analyzed participants.

For baseline, the primary outcome variables were ventral striatum (nucleus accumbens) and dorsal striatum responses to food vs nonfood cues in the postprandial state (after glucose consumption), and the main exposure variable was the percentage of total daily calories from UPF. Regression models adjusted for child age, sex, baseline BMI, total caloric intake, and gestational diabetes exposure status to account for potential recruitment bias. Two primary brain ROIs were tested, and a Bonferroni correction was applied to assess statistical significance (corrected *P* = nominal *P* × 2) to reduce the risk of false positives.

Follow-up analyses were conducted only for striatal responses that were significantly associated with UPF intake at baseline to assess whether these were associated with subsequent changes in adiposity. The primary outcomes were 1-year changes in BMI, total body fat percentage, and waist circumference (as a surrogate for central adiposity). The independent variables were baseline striatal response to food cues and baseline percentage of calories from UPFs. Linear regression models adjusted for baseline age, sex, baseline BMI, baseline total caloric intake, and gestational diabetes exposure status. The adiposity outcomes were predefined and reflect distinct adiposity traits; all are important, clinically meaningful measures. Therefore, no multiple comparison adjustment was applied. Statistical significance was defined as a 2-sided *P* < .05, with effect sizes and 95% CIs reported throughout.

To assess whether dorsal striatal response to food cues accounted for the association between baseline UPF intake and adiposity change, we used a change in estimate approach.^25^ We compared the β coefficient for UPF intake before and after adjusting for the dorsal striatal response and calculated the percentage change in the β coefficient—([β_unadjusted_ − β_adjusted_]/β_unadjusted_) × 100—in the linear regression models. Attenuation was inferred if the β coefficient was reduced by more than 10%.^25^ A formal mediation analysis was not completed due to the small sample size.

In exploratory post hoc analyses, we examined associations between the percentage of daily calories from added sugar and striatal responses to food cues adjusted for the same covariates as the primary analysis. Furthermore, to assess whether the association between UPF and striatal responses was independent of added sugar intake, we included an additional adjustment for added sugar intake to evaluate attenuation.

All independent variables, outcome variables (dorsal striatal response to food cues, UPF intake, adiposity measurements), and covariates were standardized (*z* scores) using the cohort distribution to facilitate direct comparisons between β coefficient values.

## Results

A total of 46 children of healthy weight (<85th BMI percentile)^26^ completed baseline and 1-year follow-up visits. The mean (SD) time between visits was 448.9 (106.3) days. At baseline, the mean (SD) age was 8.7 (1.1) years, 26 children (57%) were female and 20 (43%) were male, and 28 (61%) were Hispanic or Latino, 4 (9%) were Non-Hispanic Asian or Pacific Islander, 3 (7%) were Non-Hispanic Black or African American, 8 (17%) were Non-Hispanic White, and 3 (7%) were Non-Hispanic other race or ethnicity (Table 1). BMI ranged from 13.8 to 19.9, body fat percentage ranged from 12.7% to 32.2%, and waist circumference ranged from 48.0 to 81.3 cm. Participants consumed a mean (SD) of 67.7% (17.8%) of their daily calories from UPFs and had a mean (SD) total energy intake of 1730.8 (349.2) kcal/d (Table 1). At the 1-year follow-up, participants’ BMI increased by a mean of 1.0 (95% CI, 0.6-1.4), body fat percentage increased by a mean of 0.8% (95% CI, −0.2% to 1.7%), and waist circumference increased by a mean of 3.9 cm (95% CI, 2.3-5.5 cm).

**Table 1.** Baseline Characteristics of Study Participants

Characteristic	Baseline (N = 46)
Demographic characteristics	
Age, mean (SD), y	8.7 (1.1)
Sex, No. (%)	
Male	20 (43)
Female	26 (57)
Race and ethnicity, No. (%)	
Hispanic or Latino	28 (61)
Non-Hispanic Asian or Pacific Islander	4 (9)
Non-Hispanic Black or African American	3 (7)
Non-Hispanic White	8 (17)
Non-Hispanic other^a^	3 (7)
Tanner stage, No. (%)	
1	39 (85)
2	6 (13)
3	1 (2)
Dietary intake	
Calories from UPF, mean (SD), %	67.7 (17.8)
Total caloric intake, mean (SD), kcal/d	1730.8 (349.2)
Anthropometric measures	
BMI, mean (SD)	16.4 (1.4)
Body fat, mean (SD), %	19.8 (4.5)
Waist circumference, mean (SD), cm	58.8 (5.8)

Abbreviations: BMI, body mass index (calculated as weight in kilograms divided by height in meters squared); UPF, ultraprocessed food.

^a^ Non-Hispanic other comprised participants of mixed race or ethnicity.

### Baseline: Association Between UPF Consumption and Striatal Response to Food Cues

A higher percentage of calories from UPF was significantly associated with a greater dorsal striatal response to food cues (β = 0.33; 95% CI, 0.05-0.61; *P* = .02) (Figure; eTable 2 in Supplement 1). This association remained significant after adjusting for child age, sex, BMI, total caloric intake, and maternal gestational diabetes status (β = 0.34; 95% CI, 0.07-0.61; *P* = .04). UPF consumption was not associated with ventral striatum response to food cues (β = 0.16; 95% CI, −0.14 to 0.46; *P* = .30) (eTable 2 in Supplement 1).

**Figure.**
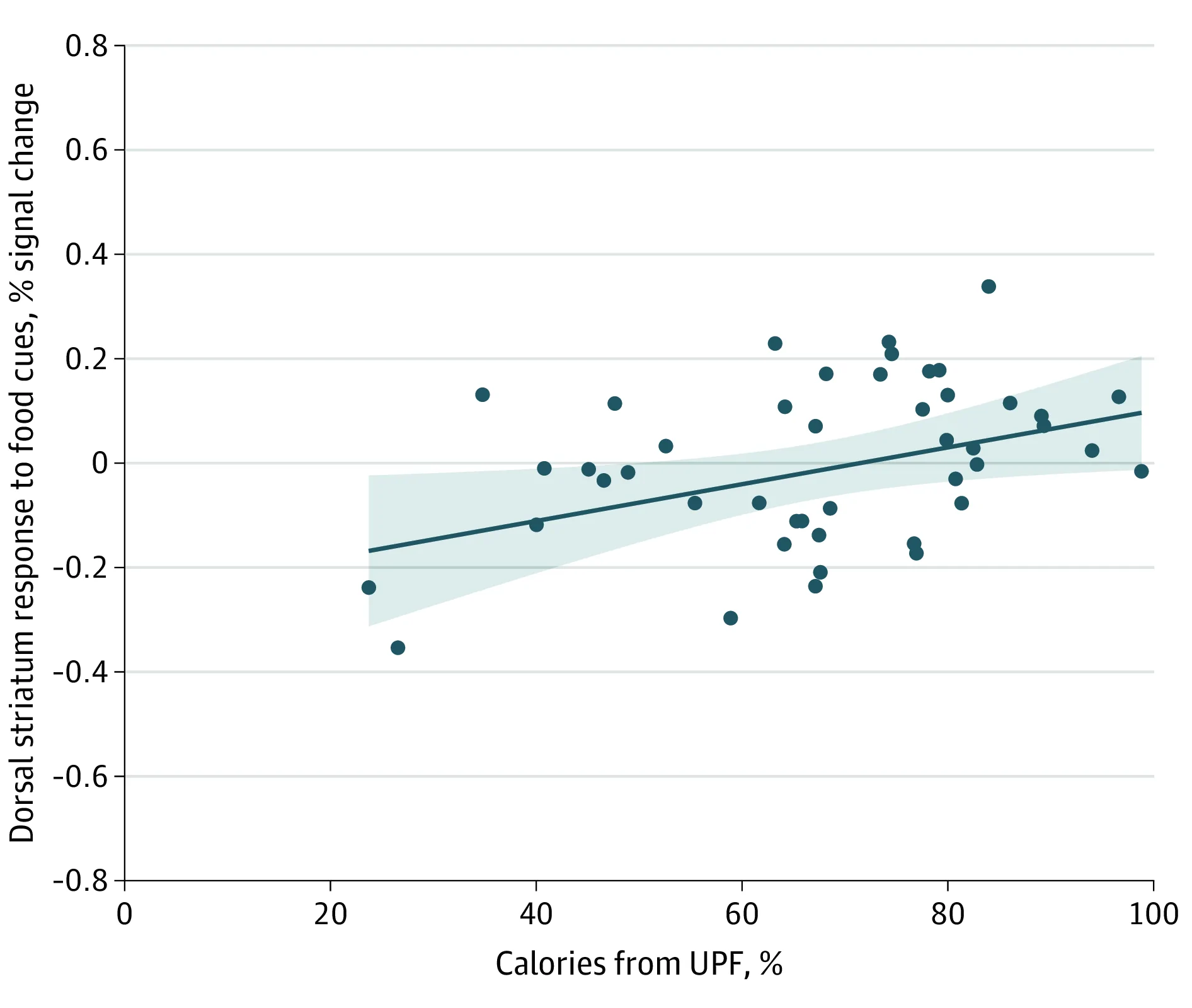
Scatterplot of the Association of Ultraprocessed Food (UPF) With Dorsal Striatal Response to Food Cues Unadjusted scatterplot showing individual participants (N = 46) with regression line with raw values for visualization purposes. Baseline percentages of daily calories from UPF vs dorsal striatum blood oxygenation level–dependent response to food vs nonfood cues in postprandial state. The shaded area indicates 95% CIs.

### Follow-Up: Striatal Responses to Food Cues and Prospective Adiposity Change

Baseline dorsal striatal response to food cues was associated with 1-year increases in BMI (β = 0.31; 95% CI, 0.03-0.59; *P* = .04) and body fat percentage (β = 0.44; 95% CI, 0.17-0.70; *P* = .002) (Table 2). Both associations remained statistically significant after adjustment for baseline age, sex, baseline BMI, baseline total caloric intake, and prenatal exposure to gestational diabetes (BMI increases: β, 0.32; 95% CI, 0.02-0.62; *P* = .04; body fat percentage: β, 0.44; 95% CI, 0.15-0.72; *P* = .005). Results were unchanged after adjusting for baseline age, sex, baseline BMI, baseline total caloric intake, and prenatal exposure to gestational diabetes.

**Table 2.** Dorsal Striatal Response and 1-Year Adiposity Gain

Outcome	Unadjusted β (95% CI)	*P* value	Adjusted β (95% CI)^a^	*P* value
Change in BMI	0.31 (0.03 to 0.59)	.04	0.32 (0.02 to 0.62)	.04
Change in body fat	0.44 (0.17 to 0.70)	.002	0.44 (0.15 to 0.72)	.005
Change in waist circumference	0.23 (−0.05 to 0.52)	.12	0.30 (−0.01 to 0.62)	.07

Abbreviation: BMI, body mass index.

^a^ Adjusted for baseline child age, sex, baseline BMI, total caloric intake, and gestational diabetes exposure status.

### UPF Consumption and Prospective Adiposity Change

Baseline UPF consumption was associated with a 1-year increase in waist circumference (β = 0.38; 95% CI, 0.11-0.66; *P* = .009), which remained significant after adjustment for covariates. Point estimates for body fat percentage and BMI were positive and of similar magnitude (body fat percentage: β = 0.29; 95% CI, 0.00-0.57; *P* = .05; BMI: β = 0.28; 95% CI, 0.00-0.57; *P* = .06) (Table 3). Within-participant changes in UPF intake and body fat percentage over the 1-year period are shown in eFigure 4 in Supplement 1.

**Table 3.** Associations Between UPF Intake and 1-Year Adiposity Gain

Outcome	Unadjusted β (95% CI)	*P* value	Adjusted β (95% CI)^a^	*P* value
Change in BMI	0.28 (−0.00 to 0.57)	.06	0.29 (0.00 to 0.57)	.05
Change in body fat	0.29 (0.01 to 0.57)	.05	0.29 (0.00 to 0.57)	.05
Change in waist circumference	0.38 (0.11 to 0.66)	.009	0.38 (0.11 to 0.67);	.01

Abbreviations: BMI, body mass index; UPF, ultraprocessed food.

^a^ Adjusted for baseline child age, sex, baseline BMI, total caloric intake, and gestational diabetes exposure status.

### Change in Estimate Approach Results

Because dorsal striatal food-cue responsivity was associated with baseline UPF intake, we examined its role in the pathway linking UPF consumption with adiposity change. In change-in-estimate analyses, inclusion of dorsal striatal responsivity attenuated the UPF coefficients for 1-year change in both body fat percentage (β, 0.29 to 0.16; 44% reduction) and BMI (β, 0.28 to 0.20; 31% reduction), each exceeding the prespecified 10% threshold for attenuation (eFigure 2 in Supplement 1).

### Post Hoc Analysis: Added Sugar and Striatal Response to Food Cues

Added sugars are a prominent constituent of many UPFs and have independently been linked with reward-related neural activity; therefore, we performed post hoc analyses to assess whether the observed association with UPF intake reflected added sugar intake specifically. Higher added sugar intake was not significantly associated with greater responses to food cues in either the dorsal striatum (β = 0.29; 95% CI, −0.02 to 0.59; *P* = .06) or the ventral striatum (β = 0.30; 95% CI, −0.01 to 0.61; *P* = .06); however, the direction was similar to that of UPFs. The percentages of calories from UPFs and from added sugar were only modestly correlated (*r* = 0.35), indicating that UPF intake captured variance beyond added sugar alone. After additional adjustment for added sugar, the association between UPF intake and dorsal striatal response was attenuated and no longer statistically significant (β = 0.28; 95% CI, −0.03 to 0.58; *P* = .07).

### Seed-to-Voxel Psychophysiological Interaction Functional Connectivity Associations With UPF

To characterize the network context of striatal responses, we conducted an exploratory analysis to assess whether UPF intake was associated with whole-brain connectivity from striatal seed regions during the food-cue task. Among children who consumed more UPFs, there was increased functional connectivity during the food-cue task between the bilateral dorsal striatum and the left precuneus and left insular cortex, and reduced functional connectivity between the dorsal striatum and the left accumbens (Table 4; eFigure 3 and eTable 3 in Supplement 1). When the ventral striatum was used as a seed region, among children who consumed more UPFs there was increased functional connectivity between the ventral striatum and the intracalcarine cortex.

**Table 4.** Psychophysiological Interaction Functional Connectivity Results With UPF Intake

Seed ROI	Brain region	Hemisphere	Coordinates^a^	Peak T	Cluster size	FWE *P* value^b^
Dorsal striatum	Precuneus cortex	Left	−18, −56, 28	4.21	1542	.001
	Insular cortex	Left	−34, −12, 12	3.92	1206	.008
	Accumbens	Left	−10, 14, −12	−5.36	809	.02
Ventral striatum	Intracalcarine cortex	Left	−16, −74, 6	5.47	4226	<.001

Abbreviations: FWE, family-wise error; ROI, region of interest; UPF, ultraprocessed food.

^a^ The Harvard-Oxford Cortical Atlas was used for cortical regions and the Harvard-Oxford Subcortical Atlas for subcortical regions. Listed regions contain the peak voxel within each cluster; other regions within each cluster are shown in eTable 3 in Supplement 1. Coordinates are in Montreal Neurological Institute space.

^b^ Cluster-level FWE-corrected *P* value.

## Discussion

In this prospective cohort study of children of healthy weight, higher habitual UPF intake was associated with greater dorsal striatal responsivity to food cues, which in turn was associated with 1-year gains in adiposity. UPF intake was associated with increased waist circumference, and adjustment for striatal responsivity attenuated the UPF coefficients for BMI and body fat change, a pattern consistent with a role for striatal reward signaling in the association between UPF consumption and adiposity gain. By integrating dietary assessment, functional neuroimaging, and longitudinal adiposity, these findings suggest that dorsal striatal reward responsivity may represent a neural pathway relevant to the association between UPF intake and adiposity gain in childhood.

Children who consumed a greater proportion of their daily calories from UPFs exhibited higher dorsal striatal activation in response to food cues. Although no prior studies have directly examined the association between habitual UPF intake and striatal responses, our findings align with evidence from studies in adults showing that higher added sugar intake is associated with greater dorsal striatal responses to food cues^27^ and with rodent models similarly demonstrating that chronic consumption of sugar-rich diets alters dorsal striatal function.^28,29^ However, added sugar is unlikely to be the sole factor associated with altered dorsal striatal function. Experimental work indicates that fat-carbohydrate combinations, characteristic of UPFs, elicit striatal reward signaling exceeding that of either macronutrient alone,^30^ and the modest correlation between added sugar and UPF intake in our sample suggests that UPF intake reflects dietary properties beyond refined sugar. Together, these converging lines of evidence support the hypothesis that the dorsal striatum is sensitive to dietary patterns characterized by highly palatable, energy-dense foods.

Higher UPF intake was associated with greater functional connectivity between the dorsal striatum and clusters in the left precuneus and insula during the food cue task. Prior fMRI studies in adults with overweight or obesity have reported increased functional connectivity in these regions during food-cue tasks.^31,32^ Given the central role of the insula in gustatory processing and the integration of food-related sensory signals,^33^ these findings raise the possibility that habitual UPF intake is associated with functional circuits involved in cue-driven food motivation. We additionally observed reduced connectivity between the dorsal and ventral striatum among children with higher UPF intake. Although exploratory, these patterns are consistent with altered organization of striatal reward circuits.

UPF intake was associated with increased activity in the dorsal, but not ventral, striatum. This dorsally weighted pattern is consistent with models of reward learning proposing that behavioral control migrates from ventral to dorsal striatal circuitry as reward-directed actions become progressively habitual and stimulus driven.^34^ Within this framework, the ventral striatum supports initial reward valuation and pavlovian (cue-driven) incentive processes, whereas recruitment of the dorsal striatum reflects the consolidation of stimulus-response associations and the transfer of behavior toward habitual, goal-independent control.^35^ This ventral-to-dorsal transition has been demonstrated in palatable food paradigms, in which repeated consumption accelerates dorsolateral-striatal habit formation.^36^ A complementary account, the incentive-sensitization theory, proposes that repeated exposure to rewarding stimuli amplifies dopamine-mediated incentive salience (wanting), rendering associated cues increasingly capable of stimulating consumption independently of their hedonic value (liking), which is instead subserved by opioid-dependent hotspots concentrated in the ventral striatum.^37,38^ Together, these frameworks suggest that greater UPF consumption in children may be associated with motivational and habit-based neural processes rather than reflecting hedonic valuation alone. The ventral striatum nonetheless contributes to UPF-related behavior through additional pathways, including its established role in delay discounting and impulsive intertemporal choice, which have been implicated in UPF consumption.^39^ Because our food-cue reactivity paradigm was not designed to probe intertemporal choice, the absence of a ventral striatal association should not be interpreted as evidence against ventral striatal involvement.

These findings are notable given our study design in which all participants were children of healthy weight at baseline and assessed in a standardized postprandial state, minimizing confounding by preexisting obesity or acute hunger. To our knowledge, this study provides the first evidence that dorsal striatal responsivity may represent a neural pathway through which diets high in UPFs are associated with prospective increases in adiposity in children of healthy weight.

### Strengths and Limitations

Our study has several strengths, including the use of the NOVA classification system to define UPF,^23,24^ integration of neuroimaging with rigorous multipass dietary assessments, and a longitudinal design with clinically relevant outcomes. Several limitations also warrant consideration. First, this study was limited by a small sample size (N = 46), which reduced statistical power to detect associations of modest magnitude. Associations between UPF intake and 1-year changes in BMI and body fat percentage were positive but did not reach statistical significance, and these estimates should be interpreted with caution. Second, fMRI studies have well-recognized concerns about reproducibility, particularly in studies with modest sample sizes.^40^ We attempted to minimize these concerns by using prospectively defined anatomical ROIs, standardized preprocessing, and rigorous quality control procedures. Nevertheless, independent replication in larger pediatric cohorts will be important. Third, our food-cue task may not capture individual food preferences and did not include concurrent behavioral ratings; we did not assess reward-related phenotypes, such as addictive-like eating or behavioral reward sensitivity, or evaluate food insecurity. These are important areas for future work. Fourth, striatal activity was assessed at a single time point, and longitudinal neuroimaging is needed to determine whether striatal alterations persist or normalize over time. Fifth, our sample of children of healthy weight reduces confounding but limits applicability to children with overweight or obesity who may show different striatal patterns. Sixth, dietary assessment is subject to reporting bias. Seventh, the observational design precludes causal inference; children with inherently greater striatal reactivity may be more prone to consuming UPFs rather than the reverse. Larger studies with repeated neuroimaging across developmental stages and weight categories will help clarify these pathways.

## Conclusions

In this cohort study of children of healthy weight, higher habitual UPF intake was associated with greater dorsal striatal responses to food cues and prospective increases in adiposity. These findings suggest that reward-related brain responses may be involved in the association between UPF consumption and adiposity gain during childhood.

## Notes

Supplement 1. **eTable 1.** Guidelines Used by Authors to Decide NOVA Level **eTable 2.** Association Between Percent Calories From Ultra-Processed Food and Striatal Response to Food Cues **eTable 3.** Brain Regions Functionally Connected to Dorsal and Ventral Striatum Seed Regions in Relation to UPF Intake During Food-Cue Viewing
eMethods.
**eFigure 1.** BrainChild Study Design and Visit Schedule **eFigure 2.** Change-in-Estimate Approach Showing Attenuation of the UPF–Body Fat Association by Dorsal Striatal Response to Food Cues **eFigure 3.** Psychophysiological Interaction (PPI) Functional Connectivity Maps Associated With Ultra-Processed Food Intake **eFigure 4.** Within-Participant Changes in Ultra-Processed Food Intake and Body Fat Percentage Over 1 Year

Supplement 2.
Data Sharing Statement
