# Supplementary material for: Ultraprocessed Food Intake, Striatal Response to Food Cues, and Adiposity Gain in Children
Source: JAMA Netw Open. 2026 Oct 23;9(10):e2640252. doi: 10.1001/jamanetworkopen.2026.40252 (PMC13695063; doi:10.1001/jamanetworkopen.2026.40252)
Supplement: Supplementary file 1 [file jamanetwopen-e2640252-s001.pdf]

Supplementary Online Content

Alves JM, Morgan E, Angelo B, et al. Ultraprocessed food intake, striatal response to food cues, and adiposity gain in children. *JAMA Netw Open.* 2026;9(10):e2640252. doi:10.1001/jamanetworkopen.2026.40252

eTable 1. Guidelines Used by Authors to Decide NOVA Level
eTable 2. Association Between Percent Calories From Ultra-Processed Food and Striatal Response to Food Cues
eTable 3. Brain Regions Functionally Connected to Dorsal and Ventral Striatum Seed Regions in Relation to UPF Intake During Food-Cue Viewing
eMethods.
eFigure 1. BrainChild Study Design and Visit Schedule
eFigure 2. Change-in-Estimate Approach Showing Attenuation of the UPF–Body Fat Association by Dorsal Striatal Response to Food Cues
eFigure 3. Psychophysiological Interaction (PPI) Functional Connectivity Maps Associated With Ultra-Processed Food Intake
eFigure 4. Within-Participant Changes in Ultra-Processed Food Intake and Body Fat Percentage Over 1 Year

This supplementary material has been provided by the authors to give readers additional information about their work.

© 2026 Alves JM et al. *JAMA Network Open.*

eTable 1. Guidelines Used by Authors to Decide NOVA Level

**Guidelines for Choosing NOVA level**
1. Check table provided by Sneed and colleagues for similar item.
2. Choose the NOVA level with the ingredient<br>with the highest number of calories~60% of total calories.
3. If item is "unknown" preparation, assume its level 4.
4. Search walmart.com or food product website for item and check<br>Ingredients if unsure.
5. Natural flavors are usually indicative that the item is ultra-processed.
6. Most commercial yogurts are ultra-processed, unless plain.

© 2026 Alves JM et al. *JAMA Network Open.*

eTable 2. Association Between Percent Calories From Ultra-Processed Food and Striatal Response to Food Cues

Region of Interest	Unadjusted	Adjusted[1]
**Ventral Striatum**	0.16 (-0.14, 0.45); *P*=.31	0.16 (-0.14, 0.46); *P*=.30
**Dorsal Striatum**	0.33 (0.05, 0.61); *P*=.02*	0.34 (0.07, 0.61); *P*=.04*

Outcome: BOLD % signal change during food vs. non-food cues
Adjustment[1]: Child age, sex, BMI, caloric intake, GDM status.
**P*<.05

eTable 3. Brain Regions Functionally Connected to Dorsal and Ventral Striatum Seed Regions in Relation to UPF Intake During Food-Cue Viewing

Seed ROI	Brain Region	Hemisphere	Cluster Size
**Dorsal Striatum**	Precuneus Cortex (15%), Posterior Cingulate Cortex (2%),	Left	1542
	Insular Cortex (2%), Left Precentral Gyrus (8%), Left Postcentral Gyrus (10%), Left Central Opercular (6%), Left Parietal Operculum Cortex (7%),	Left	1206
	Accumbens L(44%)/R(56%), Right Frontal Orbital Cortex (15%), Subcallosal Cortex (4%), L(3%)/R(2%) Putamen, Right Thalamus (1%), Right Insular Cortex (1%), Right Caudate (3%)	Left	809
**Ventral Striatum**	Intracalcarine Cortex (70%), Precuneus Cortex (22%), Lingual Gyrus L(34%)/R(9%), Posterior Cingulate Cortex (18%), Cuneal Cortex (L(32%)/R(14%), Supracalcarine Cortex L(47%)/R(10%).	Left	4226

© 2026 Alves JM et al. *JAMA Network Open.*

eMethods.

MRI Imaging Parameters: MRI scans were performed on a 3T Siemens MAGNETOM Prisma fit scanner using a 24-channel head coil. BOLD scans were acquired using a gradient echo, echo planar imaging (GE-EPI) T2*-weighted sequence with the following parameters: Repetition time (TR)=2,000 msec, echo time (TE)=25 msec, bandwidth=2230 Hz/pixel, flip angle=90°, field of view (FOV) = 220×220 mm, matrix=64×64. The BOLD scan consisted of 94 volumes of a single-shot gradient echo planar imaging sequence that had 32, 2.5 mm thick slices.

A T1 high resolution 3D Magnetization Prepared Rapid Gradient Echo Sequence was also used for multi-subject registration, it included the following parameters: (TR=2530 ms; TE=2.62 ms; bandwidth=240 Hz/pixel; flip angle= 9°; slice thickness=1mm; FOV=256×256 mm; voxel size=1mmx1mmx1mm).

fMRI Analysis

Functional MRI data were processed using FSL's fMRI Expert Analysis Tool (FEAT, version 6.00; Oxford Centre for Functional MRI of the Brain, University of Oxford, UK; http://www.fmrib.ox.ac.uk/fsl). Functional data were aligned to each participant's high-resolution anatomical T1 image. Then each image is preprocessed using the following steps: brain extraction, motion realignment, spatial smoothing with a 5 mm full-width at half-maximum Gaussian kernel, and high-pass temporal filtering (100 s). Each participant's final processed image is normalized to standard space using affine registration (FLIRT) to the Montreal Neurological Institute (MNI) template, consistent with prior studies in similarly aged samples [19,20]. Prior to analyses, region of interest masks (dorsal and ventral striatum) were created using structurally defined ROI's through the probabilistic Harvard-Oxford atlas. Each voxel was defined as a part of an ROI, based on a 50% probability. This was how anatomically defined ROIs were created for the BrainChild study.

Food and non-food cue events were modeled within a general linear model (GLM) after convolution with a canonical hemodynamic response function. Six motion parameters were

© 2026 Alves JM et al. *JAMA Network Open.*

included as nuisance regressors. First-level contrasts comparing food > non-food cues were generated for each participant and entered into group-level analyses using FMRIB's Local Analysis of Mixed Effects (FLAME1). Using FSL Featquery, the second-level contrast was completed to extract the % signal change for each participant during food images compared to nonfood images using structurally defined ROI's from each participant's native space.

<u>Preprocessing</u>

Functional and anatomical images were preprocessed using CONN's standard preprocessing pipeline, including motion correction, slice-timing correction, outlier detection, normalization to MNI space, tissue segmentation, and spatial smoothing.

Functional images were realigned to the first volume of each subject's scan using rigid-body transformations with b-spline interpolation. Slice-timing differences (Siemens interleaved acquisition) were corrected using SPM's slice-timing procedure. Outlier scans were identified based on framewise displacement (>0.9 mm) or global signal change (>5 SD) and excluded from reference image estimation.

Images were normalized to MNI space, segmented into gray matter, white matter, and CSF, resampled to 2 mm isotropic resolution, and smoothed using a 5 mm FWHM Gaussian kernel.

<u>Denoising</u>

Denoising of functional data were performed using CONN's standard denoising pipeline, which included regression of white matter and CSF signals, motion parameters and their derivatives, outlier scans, task and session effects and their derivatives, and linear trends within each functional scan.

Bandpass filtering between 0.008 and 0.09 Hz was applied to the BOLD time series.

Additionally, component-based noise correction (CompCor) estimated noise components from the white matter and CSF segmentation masks, the average BOLD signal, motion parameters, outlier scans, and the largest principal components orthogonal to the average BOLD signal. The effective degrees of freedom after denoising ranged from 15.1 to 21 (mean = 19.1).

© 2026 Alves JM et al. *JAMA Network Open.*

First-level analysis

Generalized psychophysiological interaction (gPPI) analyses were used to assess task-related changes in functional connectivity between the Control_Condition (Non-Food images) and the Task_Condition (Food images). Six Harvard–Oxford atlas regions were used to create two seed regions; the Dorsal Striatum which comprised of the bilateral Caudate and Putamen, and the bilateral Nucleus Accumbens as the Ventral Striatum.

For each seed–target pair, gPPI models included the seed BOLD signal, task regressors convolved with the canonical hemodynamic response function, and their interaction terms. Connectivity differences across conditions were estimated from the interaction term coefficients.

Group-level analysis

Group-level effects were tested using voxelwise general linear models (GLM), with first-level connectivity estimates as dependent variables and subject-level factors as predictors, including subject age, sex, and UPF intake measurements.

Random-effects analyses were conducted across subjects, with inference performed at the cluster level. Results were thresholded at $p_{FWE} < 0.05$.

fMRI Quality Control Methods: Structural and functional MRI data were evaluated for quality following image acquisition. Each subject's structural and functional images were visually inspected by trained staff for motion, magnetic susceptibility, and aliasing artifacts. Images exhibiting these artifacts were excluded from further analysis. As an additional quality control measure, functional images were preprocessed using the FSL toolbox, and absolute head motion (in millimeters) was calculated across all volumes. Subjects were excluded from the analysis if their functional images exceeded 2.0 mm of absolute motion or if FSL reported a warning indicating high levels of motion.

Dietary Quality Control: To identify implausible dietary reports, total caloric intake was regressed against participant weight for each recall, and residuals were extracted and standardized. Residual z-scores > 3 or < -3 were flagged as outliers and excluded from further

© 2026 Alves JM et al. *JAMA Network Open.*

analyses, in line with previous reports [23]. One participant had both baseline recalls removed and was therefore excluded from further analyses. Four participants had one baseline recall removed.

Processing details for NOVA classification: A reference template with over 3,300 pre-classified food items, provided by Sneed and colleagues[25], was used as the primary classification source for food items using the NOVA classification system. The template included over 3300 food-items with NOVA levels assigned based on inter-rater reliability. For food items not included in the template, four researchers assigned NOVA levels using NDSR food-item details (food ID, item name, preparation method) and external sources to identify information about each food item, including product websites. Each researcher was assigned a list of items and independently determined the NOVA levels. After the researchers assigned NOVA levels to items not found in the template provided by Sneed and colleagues, a senior researcher (J.M.A.) conducted a final review of all classifications in collaboration with the Sneed team. Similar to Sneed and colleagues[25], a uniform set of rules was used to apply the NOVA level for each food-item (See Supplemental Table 1). The percentage of total daily calories derived from ultra-processed foods (Level 4) was calculated as the primary dietary exposure variable.

Statistical Analysis:

The details of the change in estimate approach are as follows: we compared the beta coefficient for UPF consumption, before and after adjusting for dorsal striatal response to food cues and calculated the percent change in beta coefficient ($\frac{\beta_{\text{unadjusted}} - \beta_{\text{adjusted}}}{\beta_{\text{unadjusted}}} \text{x} 100$), using linear regression. Potential mediating effects were inferred if the beta coefficient was reduced by more than 10% [26].

© 2026 Alves JM et al. *JAMA Network Open.*

eFigure 1. BrainChild Study Design and Visit Schedule

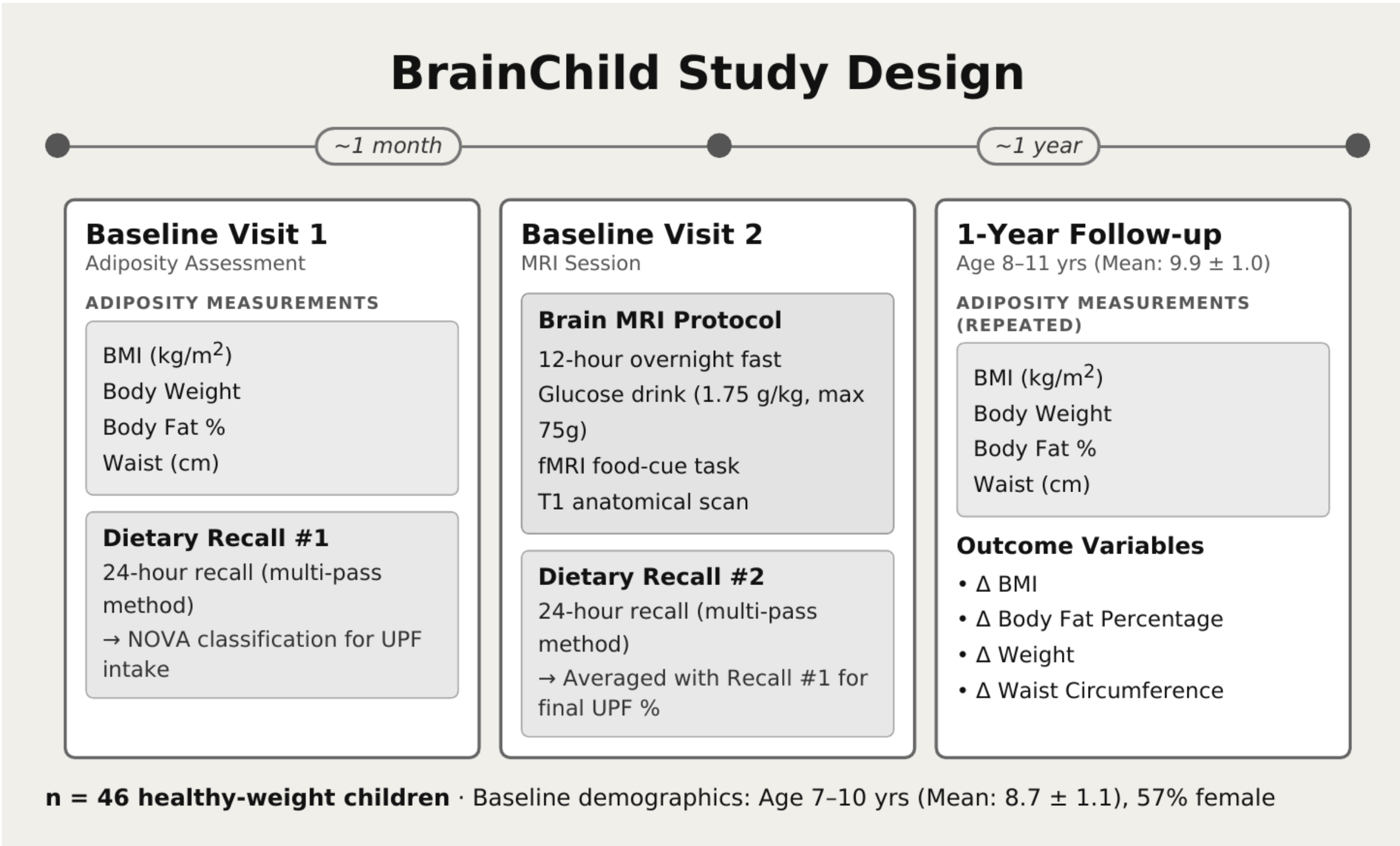

Overview of the BrainChild study visits: baseline adiposity assessment, MRI session with the food-cue task, dietary recalls, and 1-year follow-up adiposity measures.

© 2026 Alves JM et al. *JAMA Network Open.*

eFigure 2. Change-in-Estimate Approach Showing Attenuation of the UPF–Body Fat Association by Dorsal Striatal Response to Food Cues

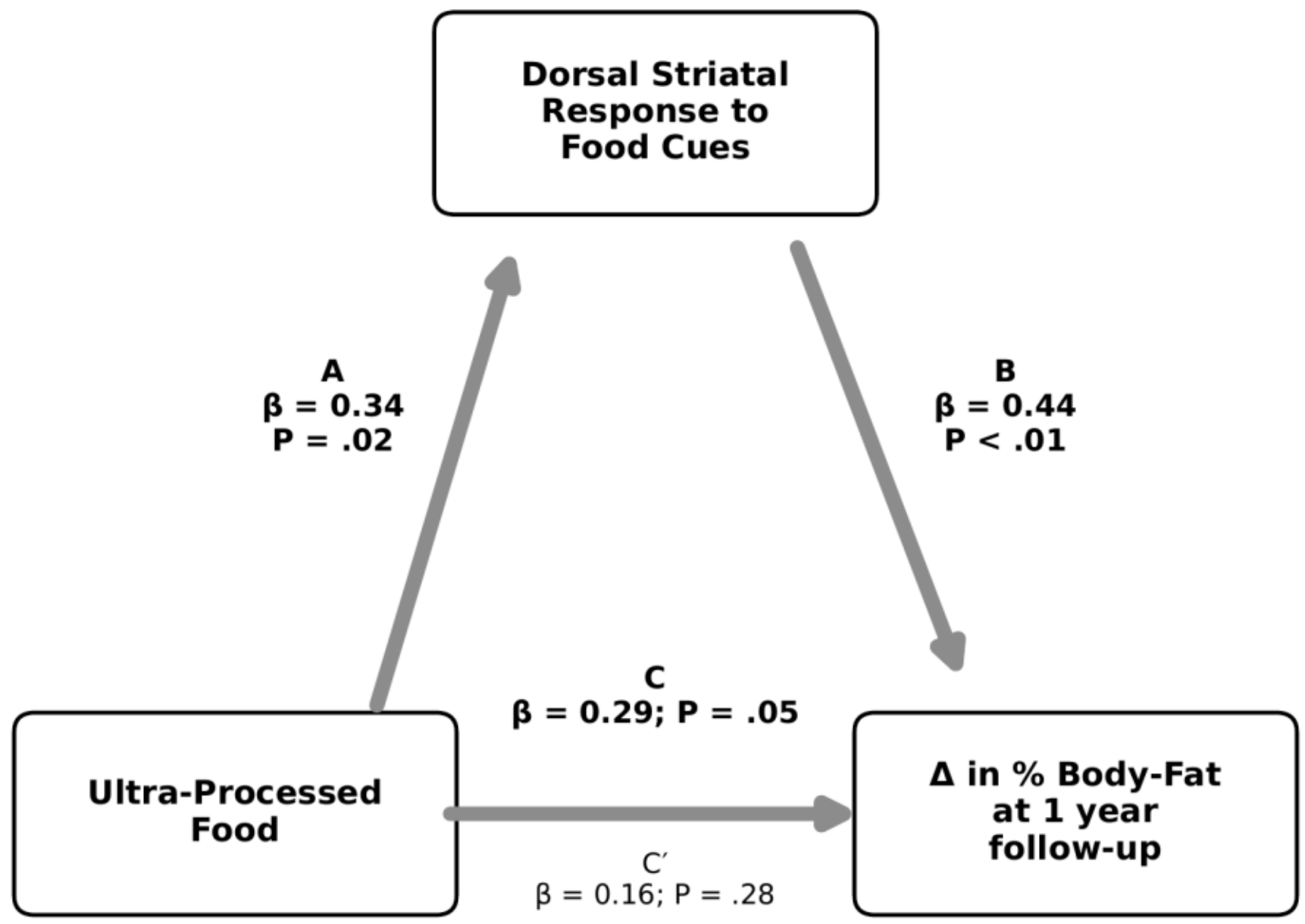

Path A: higher UPF intake associated with greater dorsal striatal response to food cues. Path B: greater dorsal striatal response associated with larger 1-year increases in body fat percentage. Path C: total UPF–body fat association; Path C′: direct association after accounting for dorsal striatal response. Dorsal striatal response accounted for 45% of the total association.

© 2026 Alves JM et al. *JAMA Network Open.*

eFigure 3. Psychophysiological Interaction (PPI) Functional Connectivity Maps Associated With Ultra-Processed Food Intake

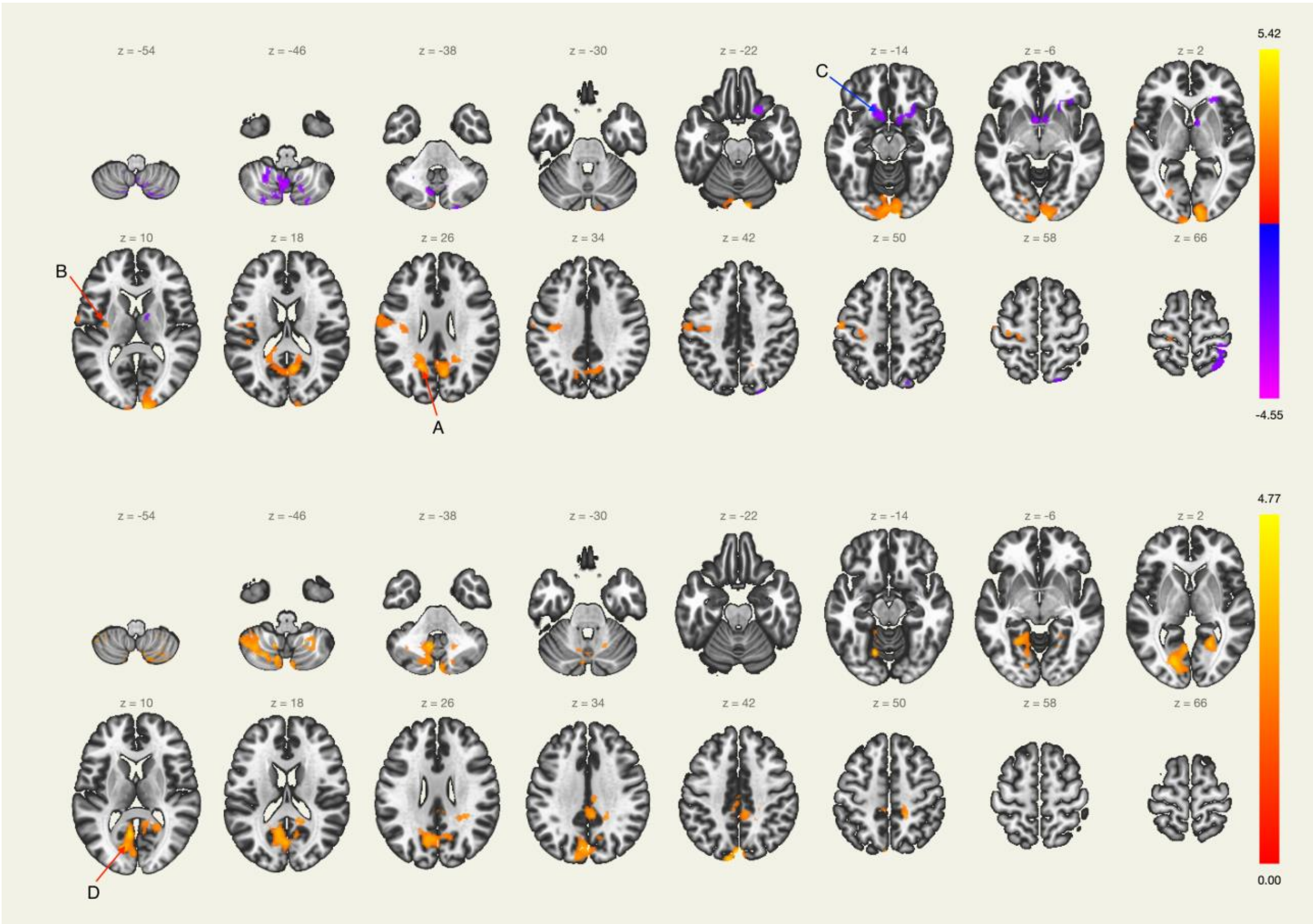

(A) Connectivity with the dorsal striatum seed during the food-cue task in children with higher UPF intake: A, precuneus cortex; B, insular cortex; C, accumbens. (B) Connectivity with the ventral striatum seed: D, intracalcarine cortex. Color bars indicate t values; warm, increased connectivity; cool, decreased connectivity.

© 2026 Alves JM et al. *JAMA Network Open.*

eFigure 4. Within-Participant Changes in Ultra-Processed Food Intake and Body Fat Percentage Over 1 Year

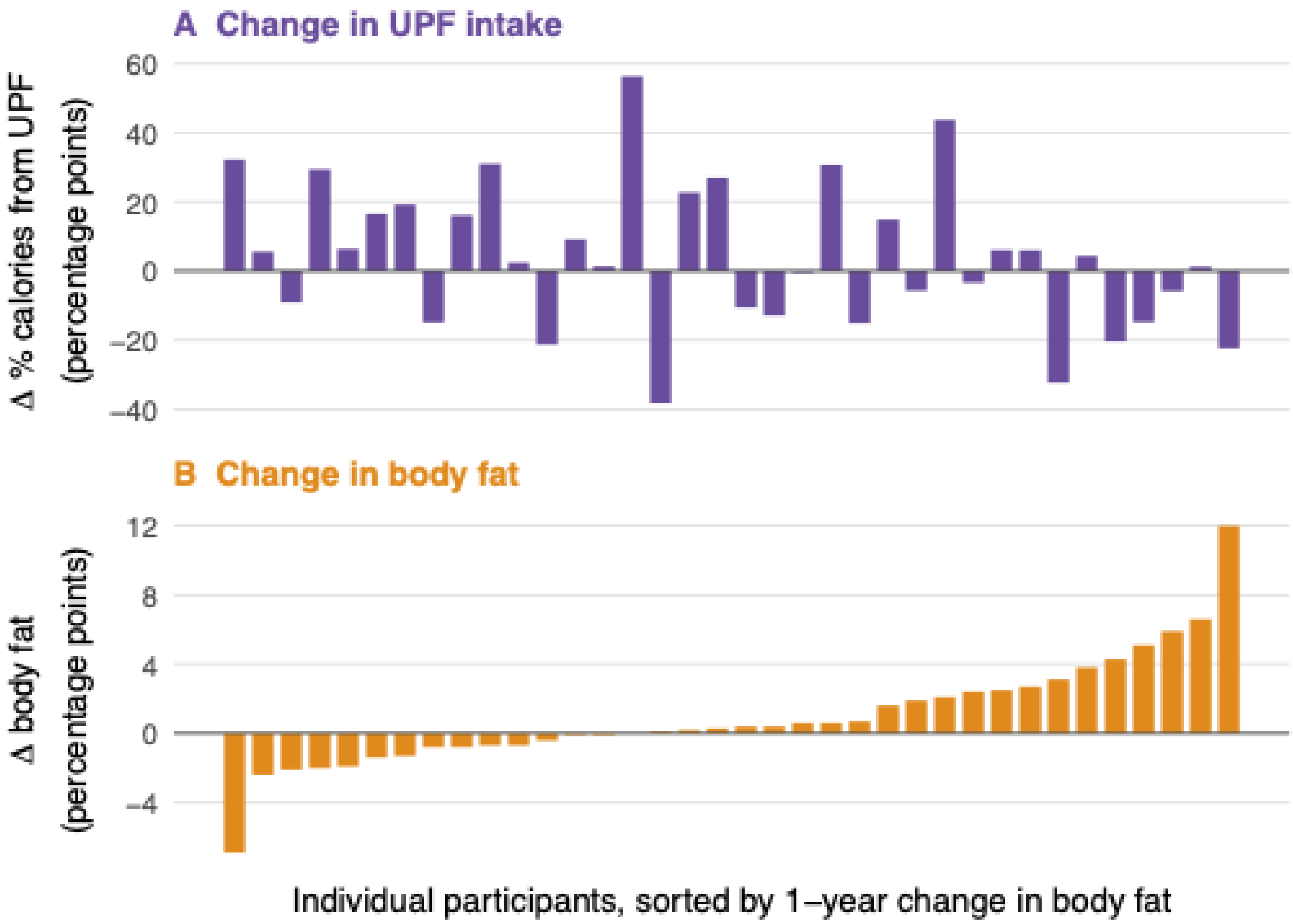

Baseline-to-1-year change for each participant, with panels in the same order (sorted by change in body fat) so within-person pairs align. (A) Change in percentage of daily calories from ultra-processed food (UPF). (B) Change in body fat percentage. The panels use independent y-axes because the measures differ greatly in scale (UPF change, ≈ −38 to +56 percentage points; body fat change, ≈ −7 to +12); a shared axis would flatten the body fat values. The figure includes the 36 of 46 participants with a 1-year dietary recall (required to compute the change in UPF); 1-year adiposity was available for all 46 participants.

© 2026 Alves JM et al. *JAMA Network Open.*
