# Supplementary material for: Ultraprocessed Food Intake, Striatal Response to Food Cues, and Adiposity Gain in Children
Source: JAMA Netw Open. 2026 Oct 23;9(10):e2640252. doi: 10.1001/jamanetworkopen.2026.40252 (PMC13695063; doi:10.1001/jamanetworkopen.2026.40252)
Supplement: Supplementary file 2 [file jamanetwopen-e2640252-s002.pdf]

Data Sharing Statement

Alves. Ultraprocessed Food Intake, Striatal Response to Food Cues, and Adiposity Gain in Children. *JAMA Netw Open*. Published October 23, 2026.
doi:10.1001/jamanetworkopen.2026.40252

Data
Data available: No

Additional Information
Explanation for why data not available: Data contains individual patient data, requests for data will be made available based on individual requests.
